# Elevated Levels of Interleukin-27 in Early Life Compromise Protective Immunity in a Mouse Model of Gram-Negative Neonatal Sepsis

**DOI:** 10.1128/IAI.00828-19

**Published:** 2020-02-20

**Authors:** Brittany G. Seman, Jordan K. Vance, Travis W. Rawson, Michelle R. Witt, Annalisa B. Huckaby, Jessica M. Povroznik, Shelby D. Bradford, Mariette Barbier, Cory M. Robinson

**Affiliations:** a Department of Microbiology, Immunology, and Cell Biology, West Virginia University School of Medicine, Morgantown, West Virginia, USA; b Vaccine Development Center, West Virginia University Health Sciences Center, Morgantown, West Virginia, USA; New York University School of Medicine

**Keywords:** IL-27 receptor, interleukin-27, WSX-1, inflammation, macrophages, neonatal, phagocytes, sepsis

## Abstract

Neonates are at increased risk for bacterial sepsis. We established that the immune-suppressive cytokine interleukin-27 (IL-27) is elevated in neonatal mice. Similarly, human cord blood-derived macrophages express IL-27 genes and secrete more cytokine than macrophages from adults. In the present work, we hypothesized that increased levels of IL-27 predispose neonatal mice to more severe infection during Gram-negative sepsis. Serum IL-27 levels continued to rise during infection. Peripheral tissue analysis revealed systemic IL-27 expression, while myeloid cell profiling identified Gr-1- and F4/80-expressing cells as the most abundant producers of IL-27 during infection. Increased IL-27 levels were consistent with increased mortality that was improved in IL-27 receptor α (IL-27Rα)^−/−^ mice that lack a functional IL-27 receptor. Infected IL-27Rα^−/−^ pups also exhibited improved weight gain and reduced morbidity. This was consistent with reduced bacterial burdens and more efficient bacterial killing by Ly6B.2^+^ myeloid cells and macrophages compared to WT neonates. Live animal imaging further supported a more severe and disseminated infection in WT neonates. This is the first report to describe the impact of elevated early-life IL-27 on the host response in a neonatal infection model while also defining the cell and tissue sources of cytokine. IL-27 is frequently associated with suppressed inflammation. In contrast, our findings demonstrate that IL-27 indirectly promotes an inflammatory cytokine response during neonatal sepsis by directly compromising control of bacteria that drive the inflammatory response. Collectively, our results suggest that IL-27 represents a therapeutic target to limit susceptibility and improve infectious outcomes in neonatal sepsis.

## Abstract (precis)

Neonates are at increased risk for bacterial sepsis. We established that the immune-suppressive cytokine interleukin-27 (IL-27) is elevated in neonatal mice. Similarly, human cord blood-derived macrophages express IL-27 genes and secrete more cytokine than macrophages from adults. In the present work, we hypothesized that increased levels of IL-27 predispose neonatal mice to more severe infection during Gram-negative sepsis. Serum IL-27 levels continued to rise during infection.

## INTRODUCTION

Neonates are highly vulnerable to bacterial infections and at increased risk of mortality. Accuracy in identifying the true global incidence of neonatal sepsis is influenced by challenges with diagnostic criteria and reliable reporting, but current estimates indicate approximately 3 million infections annually (1). In the United States alone, greater than 75,000 neonatal infections are reported annually due to sepsis (2). The rate of cases per live birth increases considerably with factors such as low birth weight and premature delivery (2). Sepsis is a leading cause of morbidity and mortality among infants in the first few days of life at any birth weight (3). This is especially true for very-low-birth-weight infants for whom sepsis also significantly increases the length of hospital stay (4, 5).

Increased susceptibility to infection in neonates is reflective of a distinct immune profile relative to adults. Phenotypic and functional differences in innate and adaptive immune function have been described in early life. In general, neonatal immunity is considered biased toward a Th2/regulatory T cell (Treg) response (6). Fewer numbers of immune cells have been found in circulation with defects reported in microbial elimination processes, antigen presentation, T cell priming, and T cell receptor repertoires (7–9). In addition, increased amounts of cytokines such as interleukin-10 (IL-10), IL-23, and IL-27 are present, further supporting an anti-inflammatory bias (10–12). This is consistent with reduced production of tumor necrosis factor alpha (TNF-α) from neonatal cells in response to Toll-like receptor (TLR) ligands compared with those from adults (13). Since adequate Th1 responses can be induced in neonates *in vivo* when given the appropriate stimulus, innate immune cells may provide signals that delay or misdirect the adaptive immune response (14). Cumulatively, these immune findings are thought to contribute to the increased susceptibility of neonates to infection.

Interleukin-27 (IL-27) is a heterodimeric cytokine that consists of the Epstein-Barr virus-induced gene 3 (EBI3) and IL-27p28 proteins (15). Engagement of the IL-27 receptor, composed of IL-27 receptor α (IL-27Rα) (also known as WSX-1 or T cell cytokine receptor [TCCR]) and gp130, predominantly activates JAK-STAT signaling (16–18). IL-27, similar to other members of the IL-12 family, was originally described as a cytokine that could drive proliferation of naive CD4^+^ T cells (19). However, mice deficient in IL-27Rα mount Th1 responses (18, 20–23). In these same animals, models of chronic disease and infection demonstrate T cell hyperactivity, suggesting that additional immune-suppressive activity may dominate (18, 21–24). Indeed, IL-27 antagonizes inflammatory T cell subsets by blocking IL-2 production and activates IL-10 production by Treg cells (25). Similarly, innate immune function is inhibited by IL-27. In macrophages, inflammatory cytokine production, inflammatory cytokine receptor expression and signaling, intracellular trafficking to lysosomes, and lysosomal acidification are negatively regulated by IL-27 (22, 26–31). This regulatory activity has been shown to compromise control of Mycobacterium tuberculosis, Staphylococcus aureus, Pseudomonas aeruginosa, and Escherichia coli (12, 26, 27, 30, 31). On the other side of the spectrum, IL-27 induces an inflammatory profile in monocytes (32). Cumulatively, this body of literature suggests that IL-27 has important immune regulatory function and opposes clearance of bacteria by macrophages. The effect of IL-27 may be cell type and context dependent, and the net influence on the complete host response in neonates has not been understood.

We have established that IL-27 is produced at elevated levels early in life. Human macrophages derived from umbilical cord blood express IL-27p28 and EBI3 genes at increased levels compared with macrophages derived from adult peripheral blood (11). This was accompanied by greater levels of secreted IL-27 protein (33). Similarly, transcript levels for IL-27 genes were increased in the spleens of neonatal and infant mice relative to adults (11). A similar pattern of IL-27 production was observed in splenic macrophages from neonatal and infant mice (11). Recently, myeloid-derived suppressor cells (MDSCs) were shown to be a significant source of IL-27, and these cells were more abundant in neonates than other age groups (12). Other reports have shown a greater abundance of MDSCs in human blood during the neonatal period (34, 35).

Considering the immune-suppressive activity of IL-27, we have hypothesized that elevated IL-27 early in life contributes to enhanced susceptibility to bacterial infection. IL-27 has been suggested as a biomarker for critically ill children and more recently declared a biomarker for early-onset neonatal sepsis (EONS) (36, 37). In the present body of work, we examined the impact of IL-27 on host protection during neonatal sepsis. We developed a murine model of EONS in response to E. coli. While group B streptococci are the leading cause of EONS overall, E. coli is responsible for the majority of deaths and is the leading cause when preterm and very-low-birth-weight babies are considered independently (3, 38). Our findings demonstrate that IL-27 compromises host control of bacteria, consistent with elevated levels of inflammatory cytokines and increased mortality.

## RESULTS

### IL-27 levels rise during neonatal sepsis.

**FIG 1:**
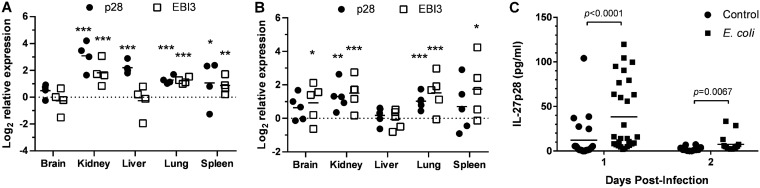
IL-27 levels rise during neonatal sepsis. Neonatal mice were subcutaneously inoculated with a target inoculum of ∼2 × 10^6^ CFU/mouse of E. coli O1:K1:H7 or PBS as a control on day 3 or 4 of life. (A, B) At 10 h (A) or 24 h (B) postinfection, the spleen, lung, kidney, liver, and brain were harvested, and RNA was isolated. The expression of IL-27p28 or EBI3 in infected tissues was determined relative to tissues from uninfected controls by real-time PCR using the formula 2^−ΔΔCt^. Each symbol represents an individual animal finding with the mean for each group displayed. An individual experiment representative of two with similar results is shown. (A, B) To assess IL-27 gene expression, nonparametric Mann-Whitney U tests were performed on ΔCt values in control and infected samples for each tissue IL-27p28 and EIB3 at 10 and 24 h. The threshold for statistical significance was set to 0.05. Data are graphically represented as the log_2_ change in gene expression relative to control. Analyses revealed statistically significant changes in p28 gene expression in infected relative to uninfected control tissues at 10 h in the kidney, liver, lung, and spleen (*P* < 0.0001, *P* < 0.0001, *P* < 0.0001, and *P* = 0.0449, respectively) and at 24 h in the kidney (*P* < 0.0004) and lung (*P* < 0.0001). Analyses revealed statistically significant differences in EBI3 gene expression in infected relative to control samples at 10 h in the kidney, lung, and spleen (*P* < 0.0001, *P* < 0.0001, and *P* = 0.0083, respectively) and at 24 h in the brain (*P* = 0.0321), kidney (*P* < 0.0001), lung (*P* < 0.0001), and spleen (*P* = 0.0321). (C) Blood was collected from infected (E. coli) and uninfected (control) mice at day 1 or 2 postinfection, and serum levels of IL-27p28 were measured by luminescent immunoassay. Statistical significance in the 95% confidence interval was determined using a Mann-Whitney U test; exact *P* values are shown.

Neonates exhibit elevated levels of IL-27 in the spleen and blood in the resting state relative to adults (11, 12). To determine if IL-27 levels continue to rise during infection and how the cytokine may impact the host response, we established a murine model of neonatal sepsis. Neonatal pups were infected with E. coli O1:K1:H7 on day 3 or 4 of life. IL-27 gene expression was measured in the lungs, livers, spleens, kidneys, and brains of mice at 10 and 24 h following infection. These time points were chosen to span a critical window during infection in the experimental model. IL-27 gene expression varied with different tissues (Fig. 1A and B). While some infected pups expressed increased levels of IL-27p28 and EBI3 transcripts in all tissues examined, in several tissues, there were some pups that did not increase IL-27 gene expression (Fig. 1A and B). At the earlier 10-h time point, the most consistent increases in IL-27p28 and EBI3 expression were observed in the lungs, spleens, and kidneys of infected neonates (Fig. 1A). Surprisingly, the latter was the site of the greatest magnitude of increase in IL-27 expression (Fig. 1). At 24 h postinfection, more pups increased expression of IL-27 genes in all tissues except the liver; however, there were still some animals that maintained lower expression levels (Fig. 1B). Importantly, because EBI3 can pair with IL-12p35 to form the IL-35 heterodimer, gene expression alone is not a strict indication of IL-27 protein levels. To further analyze IL-27 systemically during infection, serum concentrations were measured by electrochemiluminescent immunoassay on days 1 and 2 postinfection. As shown in Fig. 1C, IL-27 increased in circulation and peaked following the first day of infection in neonatal mice. While the mean IL-27 levels increased significantly more than 3-fold, the population separated into higher and lower expressers similar to the gene expression data (Fig. 1C). A 3-fold increase in IL-27 levels was maintained in infected pups relative to controls at day 2 postinfection but at reduced overall magnitude (Fig. 1C). These data demonstrate that although IL-27 levels are higher at baseline in neonates than in older populations, the levels continue to rise further during infection. Furthermore, the infected population of neonates at 24 h includes those expressing IL-27 genes and producing cytokine at higher levels as well as those that better control IL-27 production. This could have implications on the progression and outcome of the infection.

### Gr-1^+^ and F4/80^+^ cells are the most abundant IL-27 producers.

**FIG 2:**
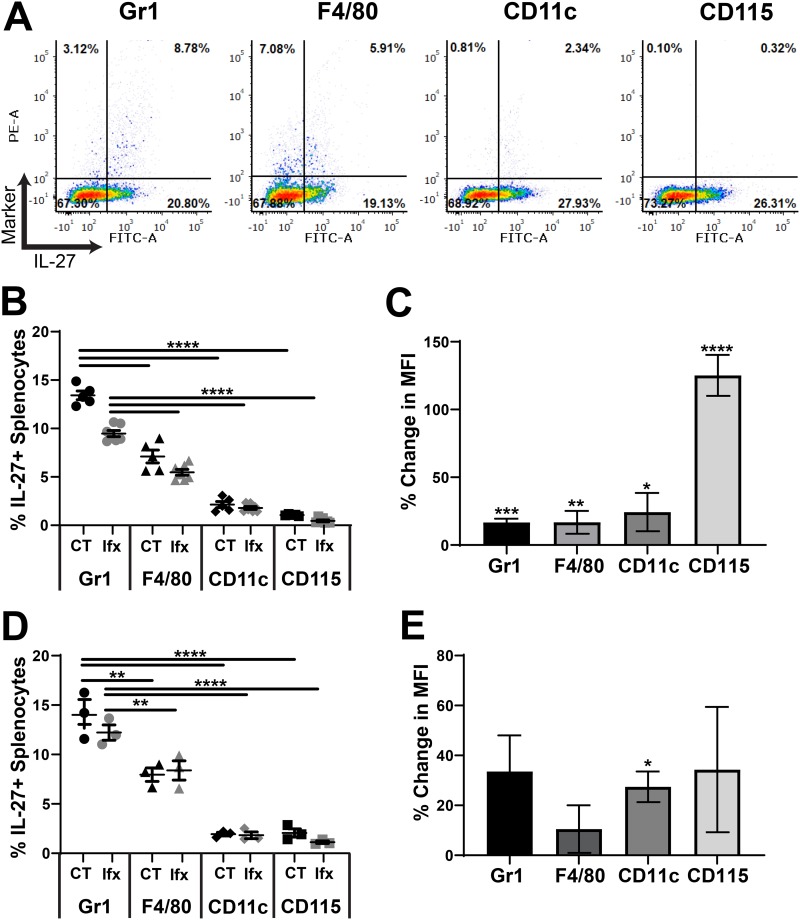
Cellular profiling of IL-27 producers in the spleen. Neonatal C57BL/6 (WT) mice were subcutaneously inoculated with a target inoculum of ∼2 × 10^6^ CFU/mouse of E. coli O1:K1:H7 or PBS as a control on day 3 or 4 of life. At 10 or 24 h postinfection, mice were sacrificed, and spleens were harvested. Single-cell suspensions of splenocytes were immunolabeled for cell surface markers Gr-1, F4/80, CD11c, or CD115 and intracellular IL-27p28. Cells were analyzed by flow cytometry. Figures represent combined results from 2 to 3 independent experiments. (A) Representative dot plots of splenocytes labeled at 10 h postinfection for the indicated marker are shown. Phycoerythrin (PE)-stained cell surface markers are represented on the *y* axis and fluorescein isothiocyanate (FITC)-stained IL-27p28 signal is represented on the *x* axis for each dot plot. (B and D) The percentage that is double positive (upper right quadrant) of the population for each cell surface marker in control (CT) and E. coli-infected (Ifx) spleens at 10 h (B) or 24 h (D) postinfection. (C and E) Percent change in mean fluorescence intensity (MFI) of fluorescein isothiocyanate (FITC) signal that corresponds to the IL-27p28 protein in the double-positive population for infected relative to control cells at 10 h (C) or 24 h (E) postinfection. (B, D) Statistical assessment was performed using a one-way analysis of variance (ANOVA) with Dunnett’s multiple comparison test. Means ± standard error are displayed. (C, E) Mean changes ± standard error in absolute values of MFI cell surface marker percentages at 10 h (C) and 24 h (E) postinfection in splenocytes were analyzed relative to a normalized baseline within the control groups using individual, unpaired *t* tests for each cell surface marker. (C) Asterisks indicate the following significant differences between infected and control splenocytes at 10 h postinfection: Gr1 (*P* = 0.0003), F4/80 (*P* = 0.0027), CD11c (*P* = 0.0237), and CD115 (*P < *0.0001). (E) At 24 h postinfection, the asterisk indicates significance for CD11c (*P* = 0.0111). Results shown for Gr-1 (*P* = 0.08) and F4/80 (*P* = 0.0689) were trending toward significance.

**FIG 3:**
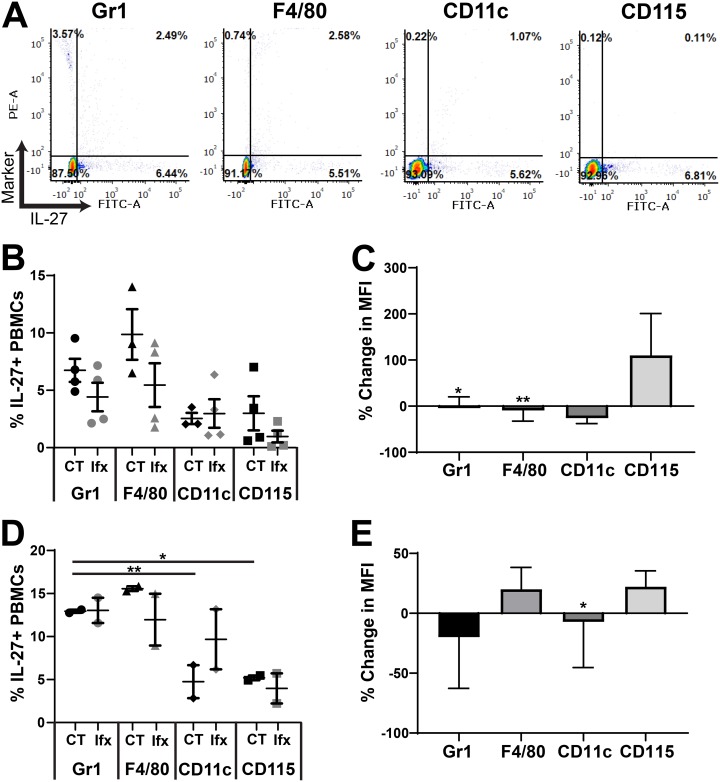
Cellular profiling of IL-27 producers in the blood. Neonatal C57BL/6 (WT) mice were subcutaneously inoculated with a target inoculum of ∼2 × 10^6^ CFU/mouse of E. coli O1:K1:H7 or PBS as a control on day 3 or 4 of life. At 10 or 24 h postinfection, mice were sacrificed, and blood was harvested and pooled for control and infected pups. PBMCs obtained by Ficoll density gradient centrifugation were immunolabeled for cell surface markers Gr-1, F4/80, CD11c, or CD115 and intracellular IL-27p28. Cells were analyzed by flow cytometry. Figures represent combined results from 2 to 3 independent experiments. (A) Representative dot plots of infected splenocytes at 10 h postinfection are labeled for the indicated marker are shown. Phycoerythrin (PE)-stained cell surface markers are represented on the *y* axis and fluorescein isothiocyanate (FITC)-stained IL-27p28 signal is represented on the *x* axis for each dot plot. (B and D) The percentage that is double positive (upper right quadrant) of the population for each cell surface marker in control (CT) and E. coli-infected (Ifx) spleens at 10 h (B) or 24 h (D) postinfection. (C and E) Percent change in mean fluorescence intensity (MFI) of fluorescein isothiocyanate (FITC) signal that corresponds to the IL-27p28 protein in the double-positive population for infected relative to control cells at 10 h (C) or 24 h (E) postinfection. (B, D) Statistical assessment was performed using a one-way analysis of variance (ANOVA) with Dunnett’s multiple-comparison test. Mean changes ± standard error are displayed. (C, E) Mean changes ± standard error in absolute values of MFI cell surface marker percentages at 10 h (C) and 24 h (E) postinfection in splenocytes were analyzed relative to a normalized baseline within the control groups using individual, unpaired *t* tests for each cell surface marker. (C) Asterisks indicate the following significant differences between infected and control splenocytes at 10 h postinfection: Gr1 (*P* = 0.0119) and F4/80 (*P* = 0.0056). Results shown for CD11c (*P* = 0.0850) at 10 h postinfection were trending toward significance. (E) At 24 h postinfection, the asterisk indicates significance for CD11c (*P* = 0.0345).

We have previously shown that MDSCs and macrophages are the dominant cellular sources of IL-27 in neonatal mice in the absence of infection (11, 12). To determine cell types that contribute to the rising IL-27 levels during infection, we profiled cells in the blood and spleens by immunofluorescent labeling and flow cytometry. Both tissues are primary sites of infection and disseminated bacteria as well as compartments with a significant population of myeloid cells. Our analysis evaluated IL-27 production in cells positive for Gr-1, F4/80, CD11c, and CD115. In the spleen and the blood, Gr-1^+^ cells were the most abundant cell type that produced IL-27 followed by a significant contribution from F4/80^+^ cells at 10 h (Fig. 2A and B, Fig. 3A and B) and 24 h (Fig. 2A and D, Fig. 3A and D, Fig. S1 and S2 in the supplemental material) postinfection. Surprisingly, there was no difference in the frequency of any IL-27-producing cell type in infected pups relative to controls in the spleen or the blood (Fig. 2 and 3, Fig. S1 and S2). However, the mean fluorescent intensity (MFI) of the IL-27 signal, indicative of the amount of IL-27 protein per cell, was increased in the spleen at 10 h in cells expressing all myeloid markers examined (Fig. 2C). CD115^+^ cells were associated with a substantial increase of nearly 100% during infection (Fig. 2C). Increased IL-27 expression was maintained at a significant level in CD11c^+^ cells at 24 h postinfection (Fig. 2E). Additionally, increased production of IL-27 in Gr-1^+^ and F4/80^+^ cells at 24 h was trending toward and nearly statistically significant (Fig. 2E). In the blood, only CD115^+^ and F4/80^+^ cells increased IL-27 production at either time point during infection, although these changes did not reach statistical significance (Fig. 3C and E). This analysis demonstrates that in the blood and spleen, myeloid cells positive for Gr-1 and F4/80 are the most abundant producers of IL-27, while cells expressing all myeloid markers examined in the spleen likely contribute to the elevated IL-27 levels observed in infected neonates.

### IL-27 promotes mortality and poor weight gain during neonatal sepsis.

**FIG 4:**
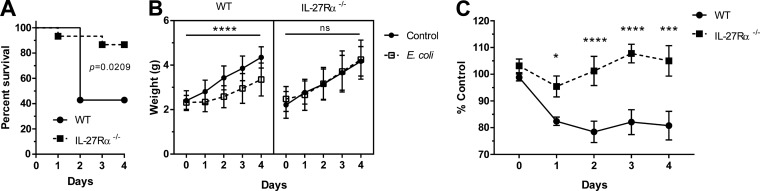
IL-27 promotes mortality and poor weight gain during neonatal sepsis. Neonatal C57BL/6 (WT) and IL-27Rα^−/−^ (KO) mice were subcutaneously inoculated with an LD_50_ dose of E. coli O1:K1:H7 or PBS as a control on day 3 or 4 of life and monitored daily for morbidity and mortality during infection. Three combined experiments for WT (*n* = 14) and KO (*n* = 15) mice infected in parallel are shown. (A) Kaplan-Meier survival curves for WT and IL-27Rα^−/−^ mice over 4 days of infection. Statistical analysis was performed using the Mantel-Cox log rank test; the exact *P* value is shown. (B) Recorded mean daily weight (g) ± standard error (SE) for control and infected mice from WT (left) and IL-27Rα^−/−^ (right) in panel A. A two-way analysis of variance (ANOVA) was used to determine statistical significance between control and E. coli-infected pups within the WT and IL-27Rα^−/−^ groups; an asterisk indicates *P* ≤ 0.0001. (C) To compare WT and KO weight gain directly, the percent change for the infected pups relative to the control pups was represented for each day. A two-way ANOVA and Bonferroni multiple-comparison test were used to determine statistical significance between WT and KO groups. *, *P ≤ *0.0141; ***, *P* ≤ 0.0001; ****, *P* ≤ 0.0003.

We further investigated the impact of elevated IL-27 levels on survival during neonatal sepsis. Mice deficient for IL-27Rα (knockout [KO]) in the C57BL/6 background do not express a functional IL-27 receptor and cannot respond to the cytokine. Morbidity and mortality were monitored over 4 days of parallel infection in KO and wild-type (WT) mice. A striking improvement in survival was observed in the absence of IL-27 signaling (Fig. 4A). Infected pups gained weight at a level comparable to uninfected controls in the KO group (Fig. 4B). In contrast, infected WT pups lagged significantly behind control pups in weight gain, an indication of morbidity (Fig. 4B). When the change in weight was expressed relative to the control pups in each group, a highly significant improvement in weight gain was evident in IL-27Rα^−/−^ neonates (Fig. 4C). This has important implications in human neonatal sepsis. The highest level of serum IL-27 at 24 h postinfection also correlates with a critical time period in disease progression (Fig. 1C and 4A). Pups that remain viable through 2 days most frequently remain viable through a 4-day infection (Fig. 4A). As such, the day 2 postinfection population is enriched for mice that are likely to remain viable through the duration of infection and may represent some survivor bias. Although we cannot definitively show that pups that deceased at day 1 or pups that were viable at day 1 and deceased at day 2 had higher circulating levels of IL-27, the trends in IL-27 gene expression, serum levels, and mortality suggest the possibility that IL-27 levels are maintained at lower concentrations in neonatal animals most likely to survive the infection (Fig. 1 and 4A). Collectively, these results indicate that IL-27 interferes with a protective host response.

### IL-27 signaling opposes host clearance of bacteria during neonatal sepsis.

**FIG 5:**
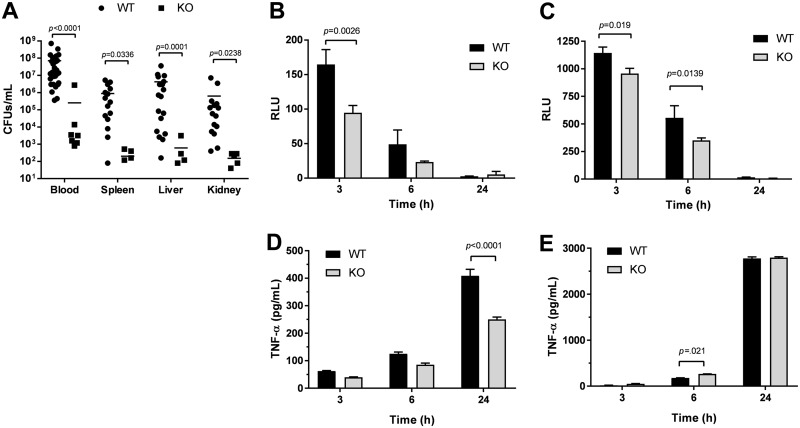
IL-27 signaling opposes host clearance of bacteria during neonatal sepsis. (A) Neonatal C57BL/6 (WT) and IL-27Rα^−/−^ (KO) mice were subcutaneously inoculated with a target inoculum of ∼2 × 10^6^ CFU/mouse of E. coli O1:K1:H7 or PBS as a control on day 3 or 4 of life. Peripheral tissues (spleen, liver, and kidney) and blood were collected at 24 h postinfection, and bacteria were enumerated by standard plate counts. (A) CFU/ml for individual animals and experimental group means are shown for two combined experiments. Statistical significance in the 95% confidence interval was determined using a Mann-Whitney test; exact *P* values are shown. (B, D) Ly6B.2^+^ cells were isolated from the spleens of C57BL/6 (WT) and IL-27Rα^−/−^ (KO) neonatal mice. (C, E) Macrophages were derived from bone marrow progenitors obtained from C57BL/6 (WT) and IL-27Rα^−/−^ (KO) neonatal mice. (B to E) Cells were seeded in 96-well plates and infected with luciferase-expressing E. coli O1:K1:H7 at a multiplicity of infection (MOI) of 50. After 1 h, the medium was replaced with fresh medium that contained gentamicin (100 μg/ml). (B, C) Relative luminescent units (RLU) were measured at 3 and 6 h postinfection. (D, E) Culture supernatants were collected at the indicated time, and TNF-α was measured by enzyme-linked immunosorbent assay (ELISA). (B to E) Mean findings ± standard error (SE) for an individual experiment representative of multiple are shown. Statistical significance in the 95% confidence interval was determined using a two-way analysis of variance (ANOVA) and Bonferroni’s multiple-comparison test; exact *P* values are shown.

To determine if improved survival in IL-27Rα^−/−^ mice is consistent with improved control of bacteria, we evaluated burdens in WT and KO mice 24 h postinfection. In the absence of IL-27 signaling, neonatal pups exhibited improved control of bacteria in the blood and all peripheral tissues examined (Fig. 5). To further explore mechanisms responsible for improved control of bacteria in IL-27Rα^−/−^ pups, we evaluated the ability of individual phagocytes to clear E. coli *in vitro*. The myeloid-restricted marker Ly6B.2 is highly expressed in neutrophils, inflammatory monocytes, and some populations of macrophages (39). Ly6B.2^+^ cells and bone marrow-derived macrophages (BMDMs) from IL-27Rα^−/−^ mice eliminated E. coli with increased efficiency early during infection (Fig. 5B and C). Similar results were obtained with F4/80^+^ cells isolated from WT and IL-27Rα^−/−^ spleens (data not shown). TNF-α levels were lower in Ly6B.2^+^ cells from IL-27Rα^−/−^ pups during *in vitro* infection and marginally higher in BMDMs at 6 h only (Fig. 5D and E). Similar results were observed for IL-6 (data not shown). Improved bacterial clearance in IL-27Rα^−/−^ phagocytes in the absence of consistently higher levels of TNF-α suggests that killing of bacteria is independent of proinflammatory cytokine production and may be a direct result of IL-27 signaling. We have previously reported that IL-27 opposes lysosomal acidification and trafficking in human macrophages with consequences to control of intracellular and extracellular bacterial growth (26, 27, 30, 31).

**FIG 6:**
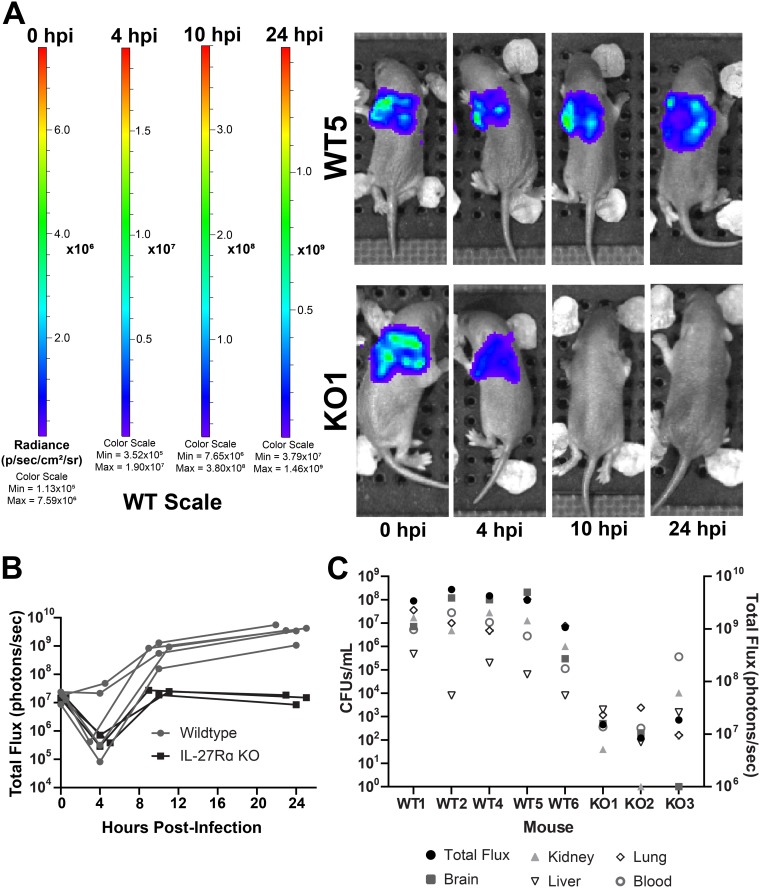
Intravital longitudinal imaging of the influence of IL-27 during neonatal sepsis. Neonatal C57BL/6 (WT) and IL-27Rα^−/−^ (KO) mice were subcutaneously inoculated with ∼2 × 10^6^ CFU/mouse of luciferase-expressing E. coli O1:K1:H7 or PBS as a control on day 4 of life in parallel. The neonatal pups were imaged longitudinally on an Ivis SpectrumCT at 0, 4, 10, and 24 h postinfection (hpi). Each mouse was tail tattooed for individual identification during imaging. Data are the result of an independent experiment (WT, *n* = 5; KO, *n* = 3) representative of two experiments with similar results. (A) Luminescent images of representative WT and KO mice at 0, 4, 10, and 24 hpi. The signal shown is on the WT scale. Colorimetric scale: low (minimum) signal is blue, intermediate signal is green, and high (maximum) signal is red. (B) Total luminescent flux in photons/second for individual mice at 0, 4, 10, and 24 hpi. (C) At 24 hpi, mice were sacrificed, and blood and peripheral tissues were collected for enumeration of bacteria by standard plate counts. Total luminescent flux (photons/second) and CFU/ml from each tissue for individually infected mice are shown.

We next wanted to examine the kinetics of bacterial clearance in real time with a focus on the critical early phase of the infection. We infected WT and KO pups with luciferase-expressing E. coli and longitudinally imaged individual mice over a 24-h period. Consistent with CFU counts in harvested tissues, there was a robust difference in luminescent signal from KO pups. As a result, WT and KO mice could not be analyzed on the same luminescence scale. The drastic difference in luminescence resulted in oversaturation of signal in WT mice placed on the KO scale (Fig. S3A). Conversely, there was an absence of signal in KO mice placed on the WT scale at 10 and 24 h postinfection, further demonstrating the significant improvement in bacterial burdens in pups that cannot respond to IL-27 (Fig. 6A). Peak luminescent signal was observed at 10 h postinfection in KO pups, indicating that bacterial replication was controlled at this point in the infection (Fig. 6B and Fig. S3B). In contrast, the luminescence measured at 10 h in WT pups was increased relative to KO pups and continued to increase through 24 h (Fig. 6B and Fig. S3B). The final signal intensity at 24 h was nearly 4 orders of magnitude higher in WT pups (Fig. 6B and C and Fig. S3B). This real-time imaging analysis also uncovered the brain as a site for high levels of bacteria in WT animals (Fig. 6C and Fig. S4). This finding was not unexpected since E. coli K1, including our strain, is a leading cause of neonatal meningitis (40, 41). However, the magnitude of difference between WT and KO pups was striking. In the absence of IL-27 signaling, there was a significant reduction in luminescent signal and CFU in the brain (Fig. 6C and Fig. S4). Overall, the change in luminescence among mice correlated with actual CFU in tissues and blood of WT and KO mice following imaging at 24 h (Fig. 6C).

### IL-27Rα^−/−^ mice exhibit reduced levels of inflammatory cytokines during infection.

**FIG 7:**
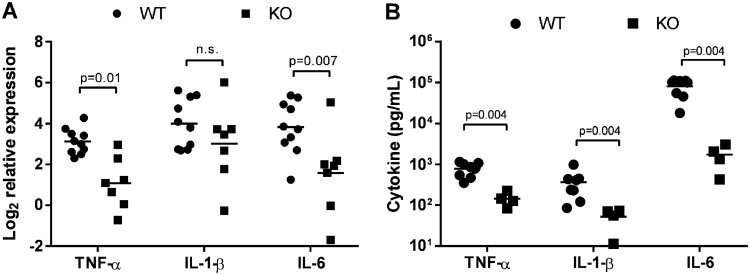
IL-27Rα^−/−^ mice exhibit reduced levels of inflammation during infection. Neonatal C57BL/6 (WT) and IL-27Rα^−/−^ (KO) mice were subcutaneously inoculated with a target inoculum of ∼2 × 10^6^ CFU/mouse of E. coli O1:K1:H7 or PBS as a control on day 3 or 4 of life. (A) Spleens were harvested 1 day postinfection, and RNA was isolated. The expression of TNF-α, IL-1, and IL-6 was determined in infected spleens relative to uninfected control spleens by real-time PCR using the formula 2^−ΔΔCt^. Individual animal findings and group means are shown for two combined experiments. Statistical significance in the 95% confidence interval was determined using individual *t* tests; exact *P* values are shown. (B) Blood was collected from mice at day 1 postinfection, and serum levels of the indicated cytokines were measured by multiplex immunoassay. Individual animal findings and group means are shown for two combined experiments. Statistical significance in the 95% confidence interval was determined using a Mann-Whitney test; exact *P* values are shown.

Failure to control bacterial replication promotes excessive and pathological inflammation during sepsis. As such, we evaluated gene expression levels of inflammatory cytokines in the spleens following 1 day of parallel infection in WT and KO neonates. This time point was chosen to evaluate pups during the critical phase; later time points would fail to include pups that succumb to infection and enrich the data set with findings from animals that exhibit improved outcomes. Gene expression levels were determined relative to uninfected controls for WT and KO mice separately. WT levels of TNF-α, IL-1, and IL-6 increased robustly in WT pups following infection, while TNF-α and IL-6 expression was significantly reduced in IL-27Rα^−/−^ pups (Fig. 7A). Although there was a trend of reduced IL-1 expression in KO pups, this finding did not reach statistical significance (Fig. 7A). The levels of serum cytokines followed a similar pattern (Fig. 7B). IL-6 levels increased dramatically in infected WT pups and were maintained at a level 2 orders of magnitude lower in KO pups, while TNF-α and IL-1 levels were reduced approximately 10-fold (Fig. 7B). IL-6 levels are increased in patients with infectious complications and are used clinically to provide a quantitative assessment of sepsis severity (42–44). Additionally, IL-6 levels correlate with the mortality rate in septic patients (45). The striking difference in IL-6 serum concentrations is reflective of peak illness in WT mice and a condition that is improved in mice that do not respond to IL-27 (46, 47).

## DISCUSSION

We have established elevated levels of the immune-suppressive cytokine IL-27 at the resting state in neonates, and we further demonstrate here that those levels continue to rise in most neonatal pups following infection that leads to sepsis. Our data regarding IL-27 serum levels are consistent with related findings in septic adult humans. Adult septic patients exhibit increased levels of IL-27 transcripts in whole blood and higher levels of serum cytokine (48). However, the absence of IL-27 data prior to infection limits our understanding of whether septic individuals are predisposed to higher IL-27 levels that constitute a risk factor for infection or if they are elevated as a consequence of infection. Our model addresses this question specifically in neonates with littermates from inbred mice. Our findings have both parallels and contrasts with adult mice that become septic following cecal ligation and puncture (CLP) for which there is IL-27-related data. In these models, splenic IL-27 transcripts rise early, peaking at 6 to 12 h, and protein levels are high in serum at 24 h (48–50). The greatest abundance of IL-27p28 transcripts at 6 h was found in the spleen and lungs (48–50). Our analysis identified the greatest increase in IL-27p28 and EBI3 transcripts during early infection in the lungs and kidneys of septic neonates. Later in the infection at 24 h, IL-27 transcripts were more widely increased across different tissues. In contrast to our neonatal data, adult mice maintain high levels of serum IL-27 through 72 h (48). Peak serum levels at 24 h during neonatal infection may be influenced by the nature of the model and critical window for survival. We are the first to profile IL-27-producing cells in the blood and tissues during septic infection of any age group. Bosmann and colleagues depleted macrophages by clodronate treatment and observed a significant decline in IL-27 in the blood during endotoxic shock (50). Our analysis of IL-27-producing myeloid cells in the spleen and blood revealed Gr-1^+^ and F4/80^+^ cells as the most abundant source of cytokine. Neither of these surface markers is exclusive to a particular cell type. Gr-1 is expressed on MDSCs, some monocyte and macrophage populations, and at a high level in granulocytes (51–53). We recently described MDSCs as a significant source of IL-27 (12), and it is tempting to speculate that these cells are a significant source of rising levels during infection. F4/80 is expressed in monocytes, macrophages, and eosinophils (54, 55). An unexpected finding was that the frequency of these cells was not increased in septic pups. However, the mean fluorescent intensity of IL-27^+^ cells demonstrated that some cells elevated their level of cytokine production at different times during infection. This was true of all myeloid populations examined in the spleen. The increased IL-27 level in CD115^+^ cells was especially dramatic at 10 h postinfection. Our cellular profiling focused on myeloid cells, which are considered the dominant cellular sources of IL-27 (56). However, we cannot rule out other cellular sources that make a contribution to the overall levels of IL-27 produced during infection. Endothelial and epithelial cells have been reported to express both IL-27 subunits (15, 56). Contributions from these cells may help to explain the high levels of IL-27 expression observed in our model in the kidney, a tissue with extensive vasculature.

The presence of IL-27 in infected neonates in our system correlates with a significant increase in bacterial burdens and mortality. We have previously reported that IL-27 interferes with lysosomal acidification and trafficking in macrophages. This promotes increased growth of intracellular and extracellular pathogens (11, 27, 30, 31). Similarly, we recently reported that MDSC-derived IL-27 opposes control of E. coli by macrophages (12). In this report, we demonstrated improved clearance of bacteria by Ly6B.2^+^ myeloid cells and bone marrow-derived macrophages from IL-27Rα^−/−^ pups. It is likely that the previously reported influence of IL-27 on lysosomal activity is directly responsible for enhanced killing of E. coli shown here (30, 31). Furthermore, since survival from sepsis in murine neonates does not depend on an intact adaptive immune system (57), the improved innate immune cell-mediated clearance of bacteria in the absence of IL-27 is likely critical to the improved mortality in those neonatal pups. Lower levels of inflammatory cytokines in IL-27Rα-deficient neonates during infection are consistent with reduced bacterial burdens. Bacteria and bacterial-derived products drive the pathological inflammatory response during sepsis. A reduced inflammatory response in the absence of IL-27 may seem counterintuitive given many literature precedents, but our results suggest that the direct influence of IL-27 on bacterial killing by phagocytes is the dominant mechanism that dictates outcomes during neonatal sepsis. This implies that the greater numbers of circulating bacteria in WT pups drive an enhanced inflammatory response due to this negative influence of IL-27 on phagocyte clearance. Similarly, reduced concentrations of inflammatory cytokines and chemokines were found in the blood in adult mice given an IL-27-neutralizing antibody during endotoxic shock and in the lungs of IL-27Rα^−/−^ adult mice during a CLP-induced impairment of secondary bacterial challenge (48, 50). Fang and colleagues demonstrated that IL-27 neutralization reduced pulmonary inflammation in a mouse model of CLP-induced lung injury (58). It is also important to consider a possible effect of IL-27 on endothelial cells. IL-27 has been implicated in the endothelial dysfunction that occurs during cardiovascular pathology central to atherosclerosis by stimulating inflammatory cytokine and chemokine expression (59). Furthermore, IL-27 increased production of IL-6 and an inflammatory chemokine cascade in human endothelial cells (60, 61). This highlights the double-edged-sword nature of IL-27. IL-27 has also been shown to activate an inflammatory response and suppress IL-10 production in monocytes (32). These cells would be expected to be significant players in the innate immune response during bacterial sepsis.

The intravital imaging analysis further supports the conclusion that IL-27 opposes bacterial clearance and allowed us to observe the rapid progression of dissemination that occurs in WT pups. To our knowledge, this is the first time bacterial sepsis has been imaged intravitally in neonatal mice. To this point, studies on sepsis in the context of lipopolysaccharide (LPS), group B streptococci, or E. coli in neonates have utilized confocal imaging of fixed tissue sections for analysis of bacterial load and inflammation (62–64). The presence of bacteria in the brain further validates our model as one that recapitulates findings clinically relevant in human neonates infected with E. coli K1 (40, 41). Our study represents a novel approach to understanding bacterial dissemination in a neonatal model relative to the host response, and drives home a direct association between IL-27 and severity of infection.

Improved infection control in adult mice lacking EBI3 or IL-27Rα occurs during both M. tuberculosis and P. aeruginosa infections or CLP-induced peritonitis (21, 22, 48, 49). However, this improved infection outcome is in contrast to other studies that demonstrate elimination of IL-27 results in marked susceptibility to infection from Trypanosoma cruzi, Trichuris muris, Leishmania major, and Toxoplasma gondii (18, 20, 23, 65, 66). The differences in infection outcome relative to IL-27 suggest a microbe and Th1- versus Th2-dependent mechanism of immunity, as well as a potential threshold of IL-27 production necessary to modulate proper immunity. Although IL-27 may serve a beneficial role in the balance of inflammatory response, in different infectious contexts, over- or underproduction of this cytokine may result in immune dysregulation and pathogen expansion.

There were some limitations to our study. Overall, the number of cells that could be obtained from the blood and spleen was limited. As a result, we could not perform more extensive profiling of IL-27-producing cells. As mentioned previously, nonmyeloid cells may contribute to the total IL-27 levels and may even be undervalued in that regard. Additionally, we have not developed an approach that allows for blood sampling from viable neonates. As such, we were unable to follow each pup for IL-27 levels and subsequent bacterial burdens or viability versus mortality. The technical ability to perform this level of analysis would further strengthen our conclusions.

In summary, our results suggest that elevated levels of IL-27 early in life predispose the host to impaired control of the pathogen burden, which is further compounded by continued increases in circulating levels of IL-27 during sepsis. These findings have enormous translational potential. On the diagnostic front, IL-27 levels in circulation may predict susceptibility to septic infection and related outcomes. Similarly, IL-27 levels may predict outcomes and guide the initiation of antibiotic therapy in neonates that appear ill. Indeed, IL-27 has been proposed as a biomarker for neonatal sepsis (37). IL-27 antagonism may also offer therapeutic potential. Our results predict that reducing IL-27 levels will promote bacterial clearance, improve host response, and reduce mortality. This approach may have prophylactic value for populations at increased risk in addition to a postinfection therapy. Currently, the only available treatment options to combat bacterial sepsis are antibiotics and supportive care (67). IL-27 may represent a targeted adjunctive therapy to augment the efficacy of antibiotics to improve survival and infection-related outcomes in neonates.

## MATERIALS AND METHODS

### Animals.

Breeding pairs of C57BL/6 (WT) or IL-27Rα-deficient (KO) mice of a C57BL/6 genetic background were purchased from Jackson Laboratories (Bar Harbor, ME) and maintained at West Virginia University School of Medicine. Male and female pups were used for experimental infection. Mice in this study were defined as neonates through 8 days of life as described previously (11, 12). Blood and tissues were collected from mice at the appropriate age by humane procedures. All procedures were approved by the West Virginia University Institutional Animal Care and Use Committees and conducted in accordance with the recommendations from the *Guide for the Care and Use of Laboratory Animals* by the National Research Council (NRC 2011) (69).

### Bioluminescent E. coli.

E. coli O1:K1:H7 (ATCC, Manassas, VA) was transformed with the pGEN-luxCDABE plasmid (Addgene catalog no. 44918) by electroporation using a MicroPulser (Bio-Rad, Hercules, CA). This plasmid contains five *lux* genes with a selectable ampicillin resistance marker (Amp^r^). To generate a stable integration, E. coli O1:K1:H7 was transformed with the pMQ-tn-PnptII-lux suicide vector (a gift by Dr. Robert Shanks, University of Pittsburgh). This plasmid contains a transposable element upstream of five *lux* genes, with a selectable ampicillin resistance marker (Amp^r^). Transformation was performed by mating with the auxotrophic strain RHO3 (68). Transformants were selected on ampicillin-supplemented LB agar and screened for a luminescent signal on a chemiluminescent imager. Luminescence was monitored through 48 h of growth and infection to assess plasmid retention. Intravital imaging was performed with E. coli-expressing luciferase from the transformed plasmid. E. coli with stably integrated *lux* genes was used in gentamicin protection assays to evaluate bacterial clearance.

### Murine sepsis infection model.

Neonatal pups at the ages of 3 or 4 days were infected subcutaneously in the scapular region with E. coli strain O1:K1:H7. The bacteria from pretitered frozen cultures were washed with phosphate-buffered saline (PBS), centrifuged at 2,000 × *g* for 5 min, and suspended in a volume of PBS equivalent to an inoculum of 50 μl/mouse. Mice were inoculated using a 28-gauge insulin needle (Covidien, Dublin, Ireland). Vehicle (PBS)-inoculated pups were identified from bacterium-infected pups using a tail snip. Survival studies were performed with an inoculum of ∼10^7^ CFU/mouse representing an approximate 50% lethal dose (LD_50_). Other experiments to evaluate infection-related parameters were performed with a target inoculum of ∼2 × 10^6^ CFU/mouse to reduce mortality so that sufficient numbers of control and infected animals could be studied in each experiment. Weights of mice were recorded immediately prior to infection and then daily thereafter. Following infection, mice were monitored twice daily for signs of morbidity. Mice exhibiting signs of morbidity (i.e., unable to right themselves, significant weight loss, and lack of movement) that met endpoint criteria were humanely euthanized. Peripheral tissues (spleen, liver, kidney, brain, and lung) isolated from pups were placed in PBS on ice. Blood was deposited in tubes that contained 5 μl of 500 mM EDTA acid (EDTA, Fisher Scientific, Fair Lawn, NJ).

### Bacterial burdens.

Peripheral tissues were homogenized in PBS using a handheld pestle motor (Kimble Chase, Vineland, NJ). Tissue homogenates and blood were serially diluted in PBS, and bacteria were enumerated by standard plate counting on tryptic soy agar (TSA; Becton, Dickinson and Company, Sparks, MD). Agar plates were incubated at 37°C overnight.

### Intracellular cytokine staining.

Spleens were crushed in a 40-μm nylon strainer. Single-cell suspensions were treated with ammonium-chloride-potassium (ACK) lysing buffer (Lonza, Walkersville, MD) to lyse red blood cells and washed in PBS that contained 10% fetal bovine serum (FBS). Blood was pooled from control or infected mice and washed in PBS. Peripheral blood mononuclear cells (PBMCs) were isolated by Ficoll (GE Healthcare Life Sciences, Chicago, IL) density gradient centrifugation at 400 × *g* for 30 min. Splenocytes and PBMCs were then treated with FcR blocking reagent (Miltenyi Biotec, Bergisch Gladbach, Germany) and a BD GolgiStop protein transport inhibitor (Becton, Dickinson, Franklin, NJ) to inhibit protein secretion. Cell surface markers were immunolabeled with anti-Gr-1 (PE rat anti-mouse Ly6G and Ly6C; BD Pharmingen, Franklin, NJ), F4/80 (anti-F4/80 PE; Miltenyi Biotec), CD11c (CD11c-PE; Miltenyi Biotec), or CD115 (CD115-PE; Miltenyi Biotec), washed, and fixed with 3% paraformaldehyde. Intracellular cytokine IL-27p28 was labeled with anti-mIL-27 (R&D Systems, Minneapolis, MN) as described previously (11). Immunolabeled cells were analyzed with a BD Fortessa flow cytometer and FCS Express (version 6; De Novo Software, Glendale, CA).

### Quantitative real-time PCR.

Spleens were homogenized in TRI reagent (Molecular Research Center, Cincinnati, OH). Using the commercial product protocol, the upper aqueous layer following phase separation was mixed with an equal volume of 75% ethanol and transferred to E.Z.N.A. RNA isolation columns (Omega Biotek, Norcross, GA). iScript cDNA synthesis reagents (Bio-Rad, Hercules, CA) were used to generate first-strand cDNA according to the manufacturer’s protocol. Real-time cycling of reactions that included cDNA diluted 15-fold from above, gene-specific primer probe sets (Applied Biosystems, Foster City, CA), and iQ Supermix (Bio-Rad) was performed in triplicate using a StepOnePlus (Applied Biosystems) real-time detection system. Gene-specific amplification was normalized to β-actin as an internal reference gene. Log_2_-transformed changes in gene expression for infected tissues relative to uninfected control tissues were determined using the formula 2^−ΔΔCt^. Thus, the data shown reflect the gene expression changes during infection.

### Cytokine measurements.

Blood was collected from mice during euthanasia at the indicated age and serum collected by standard techniques. IL-1, IL-6, and TNF-α serum levels were measured using multiplexed luminescent detection reagents according to the manufacturer’s protocol (MesoScale Discovery [MSD], Rockville, MD). Serum concentrations of IL-27p28 were measured using single-analyte luminescent detection reagents (MesoScale Discovery). Results were analyzed using MSD Discovery Workbench software. Protein standards were assayed in parallel with samples.

### *In vivo* imaging.

Neonatal pups were imaged using an Ivis SpectrumCT (PerkinElmer, Waltham, MA). Mice were infected with the bioluminescent E. coli and imaged over time for location and intensity of luminescence. To decipher between individual mice over time, tails were tattooed by use of a 28-gauge insulin needle that inserts green or black tattoo paste (Ketchum Manufacturing, Lake Luzerne, NY). Pups were anesthetized by use of an isoflurane chamber and kept under anesthesia during imaging. Luminescence signal and images were processed using Living Image 4.5 software (PerkinElmer, Waltham, MA). Briefly, signal was quantified by region-of-interest (ROI) construction around the area of luminescence in two-dimensional images. Signal was quantified in radiance units and represented as total flux (photons/second). Luminescence scales were set according to the colorimetric scale for WT or KO mice at each time point due to the profound differences in signal between the two genotypes.

### Gentamicin protection bacterial clearance assays.

To generate bone marrow-derived macrophages (BMDM), bone marrow progenitors were extracted from femurs, tibias, radius-ulna, and humerus bones of C57BL/6 or IL-27Rα^−/−^ neonatal mice in α-minimal essential medium (MEM; Corning, NY) containing 10% FBS, 2 mM glutamine, and 100 U/ml penicillin/streptomycin. The contents were strained through a 40-μm nylon strainer to remove residual tendon and ligament tissue, and erythrocytes were lysed using 0.2% sodium chloride and neutralized in 1.6% sodium chloride. Following a wash with PBS, bone marrow cells were differentiated in Dulbecco modified Eagle medium (DMEM) that contained 2 mM glutamine, 25 mM HEPES, 10% fetal calf serum (FCS), and 10% L cell-conditioned medium for 5 to 7 days at 37°C with 5% CO_2_ as described previously (12). MDSCs were cultured in DMEM that contained 2 mM glutamine, 25 mM HEPES, and 10% FCS. Ly6B.2^+^ and F4/80^+^ cells were isolated from splenocytes prepared as described above by immunomagnetic selection using Miltenyi isolation reagents (Miltenyi Biotec). BMDMs, F4/80^+^, and Ly6B.2^+^ cells were cultured with luciferase-expressing E. coli at a multiplicity of infection (MOI) of 50 for 1 h at 37°C and 5% CO_2_. The medium was then replaced with fresh medium supplemented with gentamicin (100 μg/ml), and the cultures were returned to incubation for an additional 4 h. Bacterial luminescence was measured using a Molecular Devices SpectraMax iD3 at 3 and 6 h postinfection (San Jose, CA).

### Statistical analysis.

All statistical analyses were performed using GraphPad Prism software (version 8; GraphPad, La Jolla, CA). Data were tested using the appropriate parametric or nonparametric measures, as indicated in the figure legends. The threshold for statistical significance was set to 0.05.

## Supplementary Material

Supplemental file 1

## References

[1] Fleischmann-Struzek C, Goldfarb DM, Schlattmann P, Schlapbach LJ, Reinhart K, Kissoon N 2018 The global burden of paediatric and neonatal sepsis: a systematic review. Lancet Respir Med 6:223–230. doi:10.1016/S2213-2600(18)30063-8. 29508706

[2] Simonsen KA, Anderson-Berry AL, Delair SF, Davies HD 2014 Early-onset neonatal sepsis. Clin Microbiol Rev 27:21–47. doi:10.1128/CMR.00031-13. 24396135 PMC3910904

[3] Weston EJ, Pondo T, Lewis MM, Martell-Cleary P, Morin C, Jewell B, Daily P, Apostol M, Petit S, Farley M, Lynfield R, Reingold A, Hansen NI, Stoll BJ, Shane AL, Zell E, Schrag SJ 2011 The burden of invasive early-onset neonatal sepsis in the United States, 2005–2008. Pediatr Infect Dis J 30:937–941. doi:10.1097/INF.0b013e318223bad2. 21654548 PMC3193564

[4] Różańska A, Wójkowska-Mach J, Adamski P, Borszewska-Kornacka M, Gulczyńska E, Nowiczewski M, Helwich E, Kordek A, Pawlik D, Bulanda M 2015 Infections and risk-adjusted length of stay and hospital mortality in Polish neonatology intensive care units. Int J Infect Dis 35:87–92. doi:10.1016/j.ijid.2015.04.017. 25936583

[5] Payne NR, Carpenter JH, Badger GJ, Horbar JD, Rogowski J 2004 Marginal increase in cost and excess length of stay associated with nosocomial bloodstream infections in surviving very low birth weight infants. Pediatrics 114:348–355. doi:10.1542/peds.114.2.348. 15286215

[6] Adkins B, Leclerc C, Marshall-Clarke S 2004 Neonatal adaptive immunity comes of age. Nat Rev Immunol 4:553. doi:10.1038/nri1394. 15229474

[7] Garcia AM, Fadel SA, Cao S, Sarzotti M 2000 T cell immunity in neonates. Immunol Res 22:177–190. doi:10.1385/IR:22:2-3:177. 11339354

[8] Sun CM, Fiette L, Tanguy M, Leclerc C, Lo-Man R 2003 Ontogeny and innate properties of neonatal dendritic cells. Blood 102:585–591. doi:10.1182/blood-2002-09-2966. 12663436

[9] Basha S, Surendran N, Pichichero M 2014 Immune responses in neonates. Expert Rev Clin Immunol 10:1171–1184. doi:10.1586/1744666X.2014.942288. 25088080 PMC4407563

[10] Angelone DF, Wessels MR, Coughlin M, Suter EE, Valentini P, Kalish LA, Levy O 2006 Innate immunity of the human newborn is polarized toward a high ratio of IL-6/TNF-alpha production in vitro and in vivo. Pediatr Res 60:205–209. doi:10.1203/01.pdr.0000228319.10481.ea. 16864705

[11] Kraft JD, Horzempa J, Davis C, Jung JY, Pena MM, Robinson CM 2013 Neonatal macrophages express elevated levels of interleukin-27 that oppose immune responses. Immunology 139:484–493. doi:10.1111/imm.12095. 23464355 PMC3719065

[12] Gleave Parson M, Grimmett J, Vance JK, Witt MR, Seman BG, Rawson TW, Lyda L, Labuda C, Jung JY, Bradford SD, Robinson CM 2019 Murine myeloid-derived suppressor cells are a source of elevated levels of interleukin-27 in early life and compromise control of bacterial infection. Immunol Cell Biol 97:445–456. doi:10.1111/imcb.12224. 30575117 PMC6536317

[13] Kollmann TR, Crabtree J, Rein-Weston A, Blimkie D, Thommai F, Wang XY, Lavoie PM, Furlong J, Fortuno ES III, Hajjar AM, Hawkins NR, Self SG, Wilson CB 2009 Neonatal innate TLR-mediated responses are distinct from those of adults. J Immunol 183:7150–7160. doi:10.4049/jimmunol.0901481. 19917677 PMC4556237

[14] Brook B, Harbeson D, Ben-Othman R, Viemann D, Kollmann TR 2017 Newborn susceptibility to infection vs. disease depends on complex in vivo interactions of host and pathogen. Semin Immunopathol 39:615–625. doi:10.1007/s00281-017-0651-z. 29098373

[15] Devergne O, Hummel M, Koeppen H, Le Beau MM, Nathanson EC, Kieff E, Birkenbach M 1996 A novel interleukin-12 p40-related protein induced by latent Epstein-Barr virus infection in B lymphocytes. J Virol 70:1143–1153. 8551575 10.1128/jvi.70.2.1143-1153.1996 PMC189923

[16] Pflanz S, Hibbert L, Mattson J, Rosales R, Vaisberg E, Bazan JF, Phillips JH, McClanahan TK, de Waal Malefyt R, Kastelein RA 2004 WSX-1 and glycoprotein 130 constitute a signal-transducing receptor for IL-27. J Immunol 172:2225–2231. doi:10.4049/jimmunol.172.4.2225. 14764690

[17] Hibbert L, Pflanz S, De Waal Malefyt R, Kastelein RA 2003 IL-27 and IFN-alpha signal via Stat1 and Stat3 and induce T-Bet and IL-12Rbeta2 in naive T cells. J Interferon Cytokine Res 23:513–522. doi:10.1089/10799900360708632. 14565860

[18] Villarino A, Hibbert L, Lieberman L, Wilson E, Mak T, Yoshida H, Kastelein RA, Saris C, Hunter CA 2003 The IL-27R (WSX-1) is required to suppress T cell hyperactivity during infection. Immunity 19:645–655. doi:10.1016/S1074-7613(03)00300-5. 14614852

[19] Pflanz S, Timans JC, Cheung J, Rosales R, Kanzler H, Gilbert J, Hibbert L, Churakova T, Travis M, Vaisberg E, Blumenschein WM, Mattson JD, Wagner JL, To W, Zurawski S, McClanahan TK, Gorman DM, Bazan JF, de Waal Malefyt R, Rennick D, Kastelein RA 2002 IL-27, a heterodimeric cytokine composed of EBI3 and p28 protein, induces proliferation of naive CD4+ T cells. Immunity 16:779–790. doi:10.1016/s1074-7613(02)00324-2. 12121660

[20] Hamano S, Himeno K, Miyazaki Y, Ishii K, Yamanaka A, Takeda A, Zhang M, Hisaeda H, Mak TW, Yoshimura A, Yoshida H 2003 WSX-1 is required for resistance to Trypanosoma cruzi infection by regulation of proinflammatory cytokine production. Immunity 19:657–667. doi:10.1016/S1074-7613(03)00298-X. 14614853

[21] Pearl JE, Khader SA, Solache A, Gilmartin L, Ghilardi N, deSauvage F, Cooper AM 2004 IL-27 signaling compromises control of bacterial growth in mycobacteria-infected mice. J Immunol 173:7490–7496. doi:10.4049/jimmunol.173.12.7490. 15585875

[22] Holscher C, Holscher A, Ruckerl D, Yoshimoto T, Yoshida H, Mak T, Saris C, Ehlers S 2005 The IL-27 receptor chain WSX-1 differentially regulates antibacterial immunity and survival during experimental tuberculosis. J Immunol 174:3534–3544. doi:10.4049/jimmunol.174.6.3534. 15749890

[23] Artis D, Villarino A, Silverman M, He W, Thornton EM, Mu S, Summer S, Covey TM, Huang E, Yoshida H, Koretzky G, Goldschmidt M, Wu GD, de Sauvage F, Miller HR, Saris CJ, Scott P, Hunter CA 2004 The IL-27 receptor (WSX-1) is an inhibitor of innate and adaptive elements of type 2 immunity. J Immunol 173:5626–5634. doi:10.4049/jimmunol.173.9.5626. 15494513

[24] Batten M, Li J, Yi S, Kljavin NM, Danilenko DM, Lucas S, Lee J, de Sauvage FJ, Ghilardi N 2006 Interleukin 27 limits autoimmune encephalomyelitis by suppressing the development of interleukin 17-producing T cells. Nat Immunol 7:929–936. doi:10.1038/ni1375. 16906167

[25] Awasthi A, Carrier Y, Peron JP, Bettelli E, Kamanaka M, Flavell RA, Kuchroo VK, Oukka M, Weiner HL 2007 A dominant function for interleukin 27 in generating interleukin 10-producing anti-inflammatory T cells. Nat Immunol 8:1380–1389. doi:10.1038/ni1541. 17994022

[26] Robinson CM, Jung JY, Nau GJ 2012 Interferon-gamma, tumor necrosis factor, and interleukin-18 cooperate to control growth of Mycobacterium tuberculosis in human macrophages. Cytokine 60:233–241. doi:10.1016/j.cyto.2012.06.012. 22749533 PMC3429699

[27] Robinson CM, Nau GJ 2008 Interleukin-12 and interleukin-27 regulate macrophage control of Mycobacterium tuberculosis. J Infect Dis 198:359–366. doi:10.1086/589774. 18557702 PMC2761687

[28] Robinson CM, O'Dee D, Hamilton T, Nau GJ 2010 Cytokines involved in interferon-gamma production by human macrophages. J Innate Immun 2:56–65. doi:10.1159/000247156. 20375623 PMC2943519

[29] Kalliolias GD, Gordon RA, Ivashkiv LB 2010 Suppression of TNF-alpha and IL-1 signaling identifies a mechanism of homeostatic regulation of macrophages by IL-27. J Immunol 185:7047–7056. doi:10.4049/jimmunol.1001290. 20971923 PMC3019240

[30] Jung JY, Robinson CM 2013 Interleukin-27 inhibits phagosomal acidification by blocking vacuolar ATPases. Cytokine 62:202–205. doi:10.1016/j.cyto.2013.03.010. 23557795 PMC3760007

[31] Jung JY, Robinson CM 2014 IL-12 and IL-27 regulate the phagolysosomal pathway in mycobacteria-infected human macrophages. Cell Commun Signal 12:16. doi:10.1186/1478-811X-12-16. 24618498 PMC4007735

[32] Kalliolias GD, Ivashkiv LB 2008 IL-27 activates human monocytes via STAT1 and suppresses IL-10 production but the inflammatory functions of IL-27 are abrogated by TLRs and p38. J Immunol 180:6325–6333. doi:10.4049/jimmunol.180.9.6325. 18424756

[33] Jung JY, Gleave Parson M, Kraft JD, Lyda L, Kobe B, Davis C, Robinson J, Pena MM, Robinson CM 2016 Elevated interleukin-27 levels in human neonatal macrophages regulate indoleamine dioxygenase in a STAT-1 and STAT-3-dependent manner. Immunology 149:35–47. doi:10.1111/imm.12625. 27238498 PMC4981608

[34] Rieber N, Gille C, Kostlin N, Schafer I, Spring B, Ost M, Spieles H, Kugel HA, Pfeiffer M, Heininger V, Alkhaled M, Hector A, Mays L, Kormann M, Zundel S, Fuchs J, Handgretinger R, Poets CF, Hartl D 2013 Neutrophilic myeloid-derived suppressor cells in cord blood modulate innate and adaptive immune responses. Clin Exp Immunol 174:45–52. doi:10.1111/cei.12143. 23701226 PMC3784212

[35] Schwarz J, Scheckenbach V, Kugel H, Spring B, Pagel J, Härtel C, Pauluschke-Fröhlich J, Peter A, Poets CF, Gille C, Köstlin N 2018 Granulocytic myeloid-derived suppressor cells (GR-MDSC) accumulate in cord blood of preterm infants and remain elevated during the neonatal period. Clin Exp Immunol 191:328–337. doi:10.1111/cei.13059. 28963753 PMC5801499

[36] Wong HR, Cvijanovich NZ, Hall M, Allen GL, Thomas NJ, Freishtat RJ, Anas N, Meyer K, Checchia PA, Lin R, Bigham MT, Sen A, Nowak J, Quasney M, Henricksen JW, Chopra A, Banschbach S, Beckman E, Harmon K, Lahni P, Shanley TP 2012 Interleukin-27 is a novel candidate diagnostic biomarker for bacterial infection in critically ill children. Crit Care 16:R213. doi:10.1186/cc11847. 23107287 PMC3682317

[37] He Y, Du W, Jiang H, Ai Q, Feng J, Liu Z, Yu J 2017 Multiplex cytokine profiling identifies interleukin-27 as a novel biomarker for neonatal early onset sepsis. Shock 47:140–147. doi:10.1097/SHK.0000000000000753. 27648693

[38] Hornik CP, Fort P, Clark RH, Watt K, Benjamin DK Jr, Smith PB, Manzoni P, Jacqz-Aigrain E, Kaguelidou F, Cohen-Wolkowiez M 2012 Early and late onset sepsis in very-low-birth-weight infants from a large group of neonatal intensive care units. Early Hum Dev 88(Suppl 2):S69–S74. doi:10.1016/S0378-3782(12)70019-1. 22633519 PMC3513766

[39] Rosas M, Thomas B, Stacey M, Gordon S, Taylor PR 2010 The myeloid 7/4-antigen defines recently generated inflammatory macrophages and is synonymous with Ly-6B. J Leukoc Biol 88:169–180. doi:10.1189/jlb.0809548. 20400676 PMC2892525

[40] Glode MP, Sutton A, Robbins JB, McCracken GH, Gotschlich EC, Kaijser B, Hanson LA 1977 Neonatal meningitis due of Escherichia coli K1. J Infect Dis 136:S93–S97. doi:10.1093/infdis/136.supplement.s93. 330780

[41] Yao Y, Xie Y, Kim KS 2006 Genomic comparison of Escherichia coli K1 strains isolated from the cerebrospinal fluid of patients with meningitis. Infect Immun 74:2196–2206. doi:10.1128/IAI.74.4.2196-2206.2006. 16552050 PMC1418925

[42] Du B, Pan J, Chen D, Li Y 2003 Serum procalcitonin and interleukin-6 levels may help to differentiate systemic inflammatory response of infectious and non-infectious origin. Chin Med J (Engl) 116:538–542. 12875718

[43] Fan SL, Miller NS, Lee J, Remick DG 2016 Diagnosing sepsis – the role of laboratory medicine. Clin Chim Acta 460:203–210. doi:10.1016/j.cca.2016.07.002. 27387712 PMC4980259

[44] Shane AL, Sánchez PJ, Stoll BJ 2017 Neonatal sepsis. Lancet 390:1770–1780. doi:10.1016/S0140-6736(17)31002-4. 28434651

[45] Kellum JA, Kong L, Fink MP, Weissfeld LA, Yealy DM, Pinsky MR, Fine J, Krichevsky A, Delude RL, Angus DC 2007 Understanding the inflammatory cytokine response in pneumonia and sepsis: results of the Genetic and Inflammatory Markers of Sepsis (GenIMS) Study. Arch Intern Med 167:1655–1663. doi:10.1001/archinte.167.15.1655. 17698689 PMC4495652

[46] Ng PC, Li K, Wong RP, Chui K, Wong E, Li G, Fok TF 2003 Proinflammatory and anti-inflammatory cytokine responses in preterm infants with systemic infections. Arch Dis Child Fetal Neonatal Ed 88:F209–F213. doi:10.1136/fn.88.3.f209. 12719394 PMC1721542

[47] Procianoy RS, Silveira RC 2004 The role of sample collection timing on interleukin-6 levels in early-onset neonatal sepsis. J Pediatr (Rio J) 80:407–410. doi:10.2223/JPED.1226. 15505737

[48] Cao J, Xu F, Lin S, Song Z, Zhang L, Luo P, Xu H, Li D, Zheng K, Ren G, Yin Y 2014 IL-27 controls sepsis-induced impairment of lung antibacterial host defence. Thorax 69:926–937. doi:10.1136/thoraxjnl-2014-205777. 25074706

[49] Wirtz S, Tubbe I, Galle PR, Schild HJ, Birkenbach M, Blumberg RS, Neurath MF 2006 Protection from lethal septic peritonitis by neutralizing the biological function of interleukin 27. J Exp Med 203:1875–1881. doi:10.1084/jem.20060471. 16880260 PMC2118378

[50] Bosmann M, Russkamp NF, Strobl B, Roewe J, Balouzian L, Pache F, Radsak MP, van Rooijen N, Zetoune FS, Sarma JV, Nunez G, Muller M, Murray PJ, Ward PA 2014 Interruption of macrophage-derived IL-27(p28) production by IL-10 during sepsis requires STAT3 but not SOCS3. J Immunol 193:5668–5677. doi:10.4049/jimmunol.1302280. 25348624 PMC4239188

[51] Gabrilovich DI, Nagaraj S 2009 Myeloid-derived suppressor cells as regulators of the immune system. Nat Rev Immunol 9:162–174. doi:10.1038/nri2506. 19197294 PMC2828349

[52] Hammond MD, Ai Y, Sansing LH 2012 Gr1+ macrophages and dendritic cells dominate the inflammatory infiltrate 12 hours after experimental intracerebral hemorrhage. Transl Stroke Res 3:s125–s131. doi:10.1007/s12975-012-0174-9. 23259009 PMC3524269

[53] Fleming TJ, Fleming ML, Malek TR 1993 Selective expression of Ly-6G on myeloid lineage cells in mouse bone marrow. RB6-8C5 mAb to granulocyte-differentiation antigen (Gr-1) detects members of the Ly-6 family. J Immunol 151:2399–2408. 8360469

[54] Dos Anjos Cassado A 2017 F4/80 as a major macrophage marker: the case of the peritoneum and spleen. Results Probl Cell Differ 62:161–179. doi:10.1007/978-3-319-54090-0_7. 28455709

[55] McGarry MP, Stewart CC 1991 Murine eosinophil granulocytes bind the murine macrophage-monocyte specific monoclonal antibody F4/80. J Leukoc Biol 50:471–478. doi:10.1002/jlb.50.5.471. 1721083

[56] Hall AO, Silver JS, Hunter CA 2012 The immunobiology of IL-27. Adv Immunol 115:1–44. doi:10.1016/B978-0-12-394299-9.00001-1. 22608254

[57] Wynn JL, Scumpia PO, Winfield RD, Delano MJ, Kelly-Scumpia K, Barker T, Ungaro R, Levy O, Moldawer LL 2008 Defective innate immunity predisposes murine neonates to poor sepsis outcome but is reversed by TLR agonists. Blood 112:1750–1758. doi:10.1182/blood-2008-01-130500. 18591384 PMC2518883

[58] Xu F, Liu Q, Lin S, Shen N, Yin Y, Cao J 2013 IL-27 is elevated in acute lung injury and mediates inflammation. J Clin Immunol 33:1257–1268. doi:10.1007/s10875-013-9923-0. 23842867 PMC7102048

[59] Dorosz SA, Ginolhac A, Kahne T, Naumann M, Sauter T, Salsmann A, Bueb JL 2015 Role of calprotectin as a modulator of the IL27-mediated proinflammatory effect on endothelial cells. Mediators Inflamm 2015:737310. doi:10.1155/2015/737310. 26663990 PMC4664814

[60] Feng XM, Chen XL, Liu N, Chen Z, Zhou YL, Han ZB, Zhang L, Han ZC 2007 Interleukin-27 upregulates major histocompatibility complex class II expression in primary human endothelial cells through induction of major histocompatibility complex class II transactivator. Hum Immunol 68:965–972. doi:10.1016/j.humimm.2007.10.004. 18191724

[61] Qiu HN, Liu B, Liu W, Liu S 2016 Interleukin-27 enhances TNF-alpha-mediated activation of human coronary artery endothelial cells. Mol Cell Biochem 411:1–10. doi:10.1007/s11010-015-2563-3. 26386872

[62] Lieblein-Boff JC, McKim DB, Shea DT, Wei P, Deng Z, Sawicki C, Quan N, Bilbo SD, Bailey MT, McTigue DM, Godbout JP 2013 Neonatal E. coli infection causes neuro-behavioral deficits associated with hypomyelination and neuronal sequestration of iron. J Neurosci 33:16334–16345. doi:10.1523/JNEUROSCI.0708-13.2013. 24107964 PMC3792468

[63] Cardoso FL, Herz J, Fernandes A, Rocha J, Sepodes B, Brito MA, McGavern DB, Brites D 2015 Systemic inflammation in early neonatal mice induces transient and lasting neurodegenerative effects. J Neuroinflammation 12:82. doi:10.1186/s12974-015-0299-3. 25924675 PMC4440597

[64] Andrade EB, Magalhaes A, Puga A, Costa M, Bravo J, Portugal CC, Ribeiro A, Correia-Neves M, Faustino A, Firon A, Trieu-Cuot P, Summavielle T, Ferreira P 2018 A mouse model reproducing the pathophysiology of neonatal group B streptococcal infection. Nat Commun 9:3138. doi:10.1038/s41467-018-05492-y. 30087335 PMC6081475

[65] Artis D, Johnson LM, Joyce K, Saris C, Villarino A, Hunter CA, Scott P 2004 Cutting edge: early IL-4 production governs the requirement for IL-27-WSX-1 signaling in the development of protective Th1 cytokine responses following Leishmania major infection. J Immunol 172:4672–4675. doi:10.4049/jimmunol.172.8.4672. 15067040

[66] Robinson KM, Lee B, Scheller EV, Mandalapu S, Enelow RI, Kolls JK, Alcorn JF 2015 The role of IL-27 in susceptibility to post-influenza Staphylococcus aureus pneumonia. Respir Res 16:10. doi:10.1186/s12931-015-0168-8. 25651926 PMC4324414

[67] The INIS Study Collaborative Group. 2008 The INIS Study. International Neonatal Immunotherapy Study: non-specific intravenous immunoglobulin therapy for suspected or proven neonatal sepsis: an international, placebo controlled, multicentre randomised trial. BMC Pregnancy Childbirth 8:52. doi:10.1186/1471-2393-8-52. 19063731 PMC2626572

[68] Lopez CM, Rholl DA, Trunck LA, Schweizer HP 2009 Versatile dual-technology system for markerless allele replacement in Burkholderia pseudomallei. Appl Environ Microbiol 75:6496–6503. doi:10.1128/AEM.01669-09. 19700544 PMC2765137

[69] National Research Council. 2011 Guide for the care and use of laboratory animals, 8th ed National Academies Press, Washington, DC.

