# Supplementary material for: Elevated Levels of Interleukin-27 in Early Life Compromise Protective Immunity in a Mouse Model of Gram-Negative Neonatal Sepsis
Source: Infect Immun. 2020 Feb 20;88(3):e00828-19. doi: 10.1128/IAI.00828-19 (PMC7035946; doi:10.1128/IAI.00828-19)
Supplement: Supplemental file 1 [file zii999093000s1.pdf]

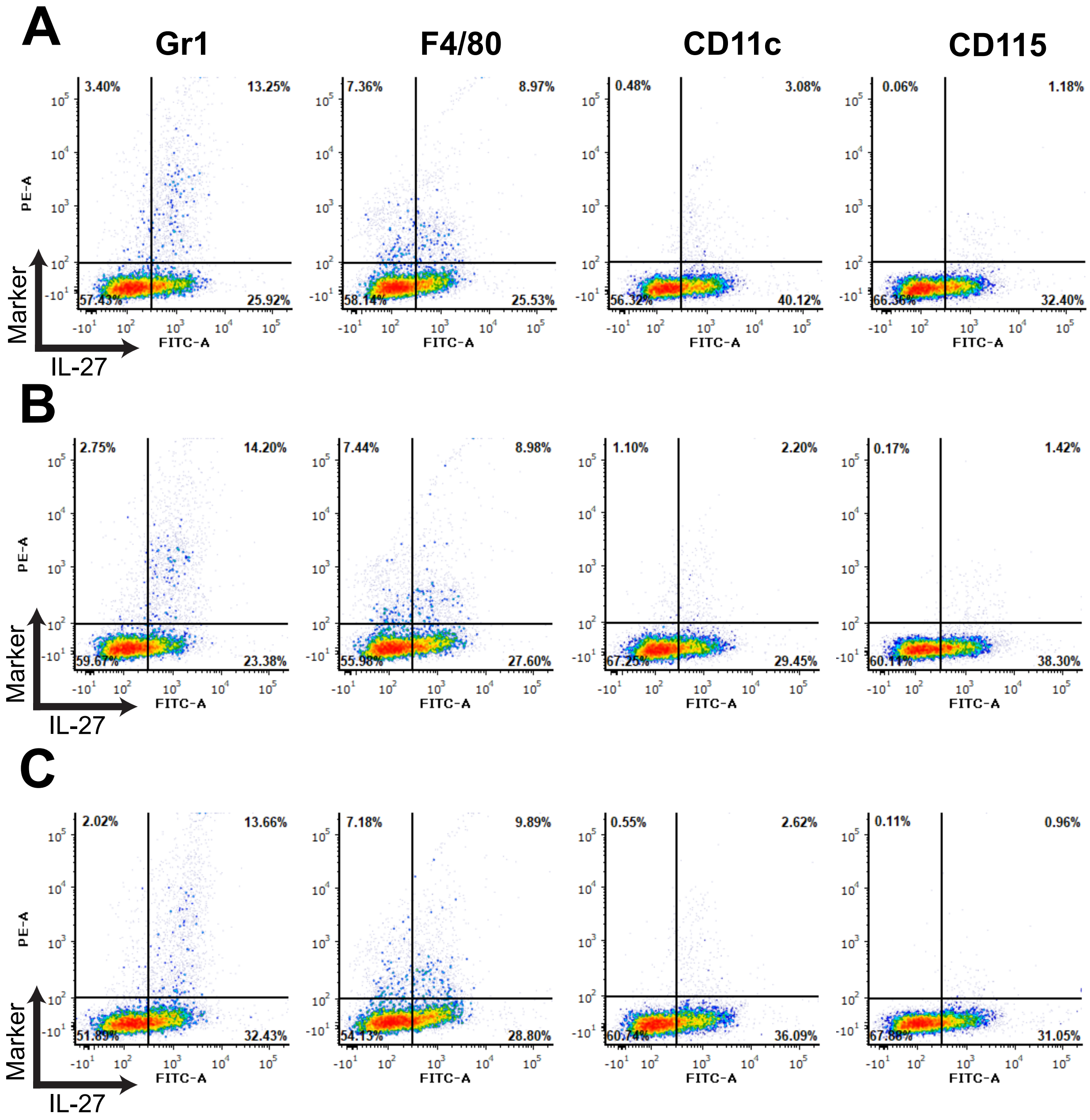
A
Gr1
F4/80
CD11c
CD115
3.40%
13.25%
57.43%
25.92%
7.36%
8.97%
58.14%
25.53%
0.48%
3.08%
56.32%
40.12%
0.06%
1.18%
66.36%
32.40%
B
2.75%
14.20%
59.67%
23.38%
7.44%
8.98%
55.98%
27.60%
1.10%
2.20%
67.25%
29.45%
0.17%
1.42%
60.11%
38.30%
C
2.02%
13.66%
51.89%
32.43%
7.18%
9.89%
54.13%
28.80%
0.55%
2.62%
60.74%
36.09%
0.11%
0.96%
67.88%
31.05%
PE-A
FITC-A
Marker
IL-27

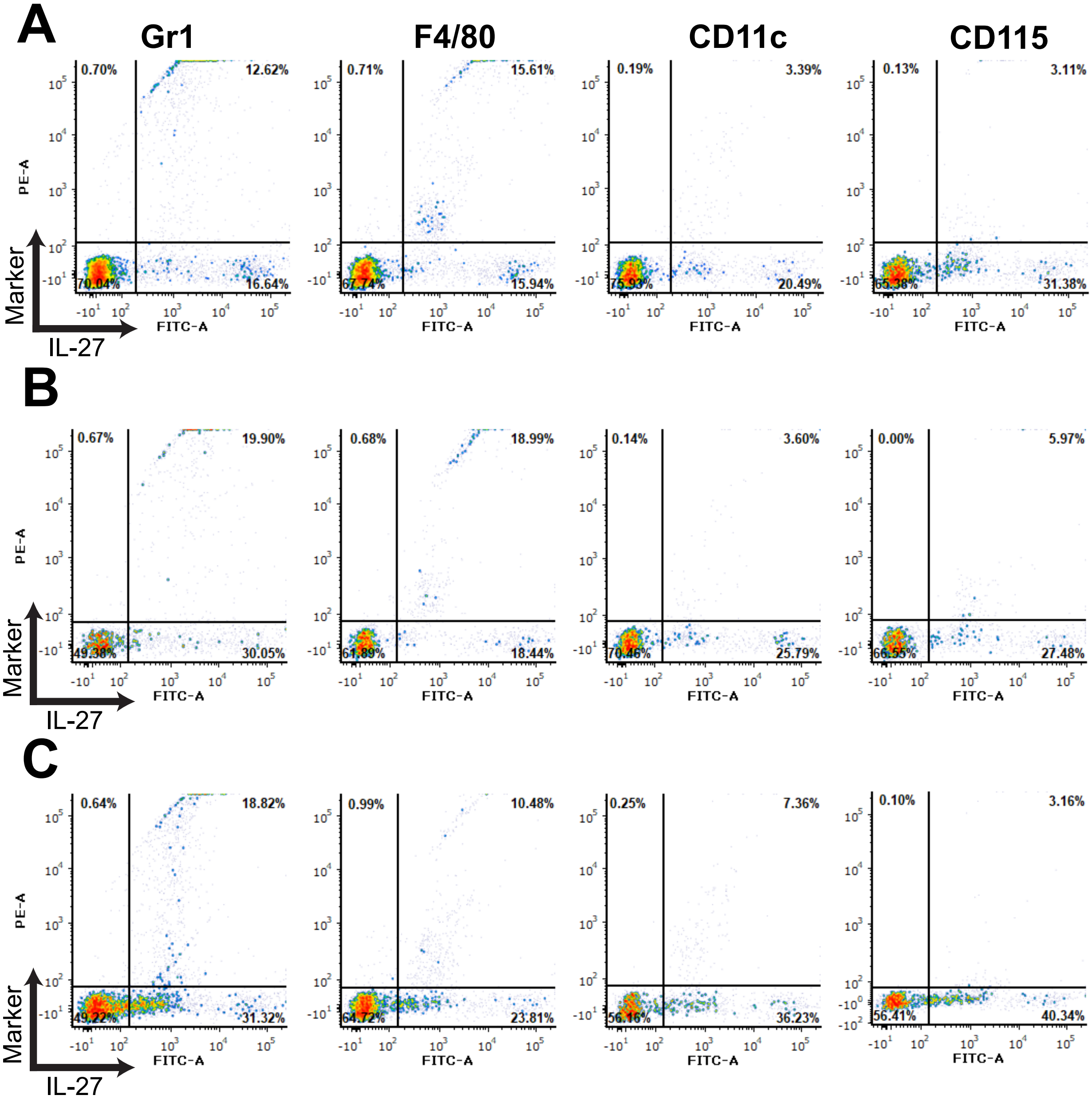
A
B
C
Gr1
F4/80
CD11c
CD115
Marker
IL-27
PE-A
FITC-A
0.70%
12.62%
70.04%
16.64%
0.71%
15.61%
67.74%
15.94%
0.19%
3.39%
75.93%
20.49%
0.13%
3.11%
65.38%
31.38%
0.67%
19.90%
49.38%
30.05%
0.68%
18.99%
61.89%
18.44%
0.14%
3.60%
70.46%
25.79%
0.00%
5.97%
66.55%
27.48%
0.64%
18.82%
49.22%
31.32%
0.99%
10.48%
64.72%
23.81%
0.25%
7.36%
56.16%
36.23%
0.10%
3.16%
56.41%
40.34%

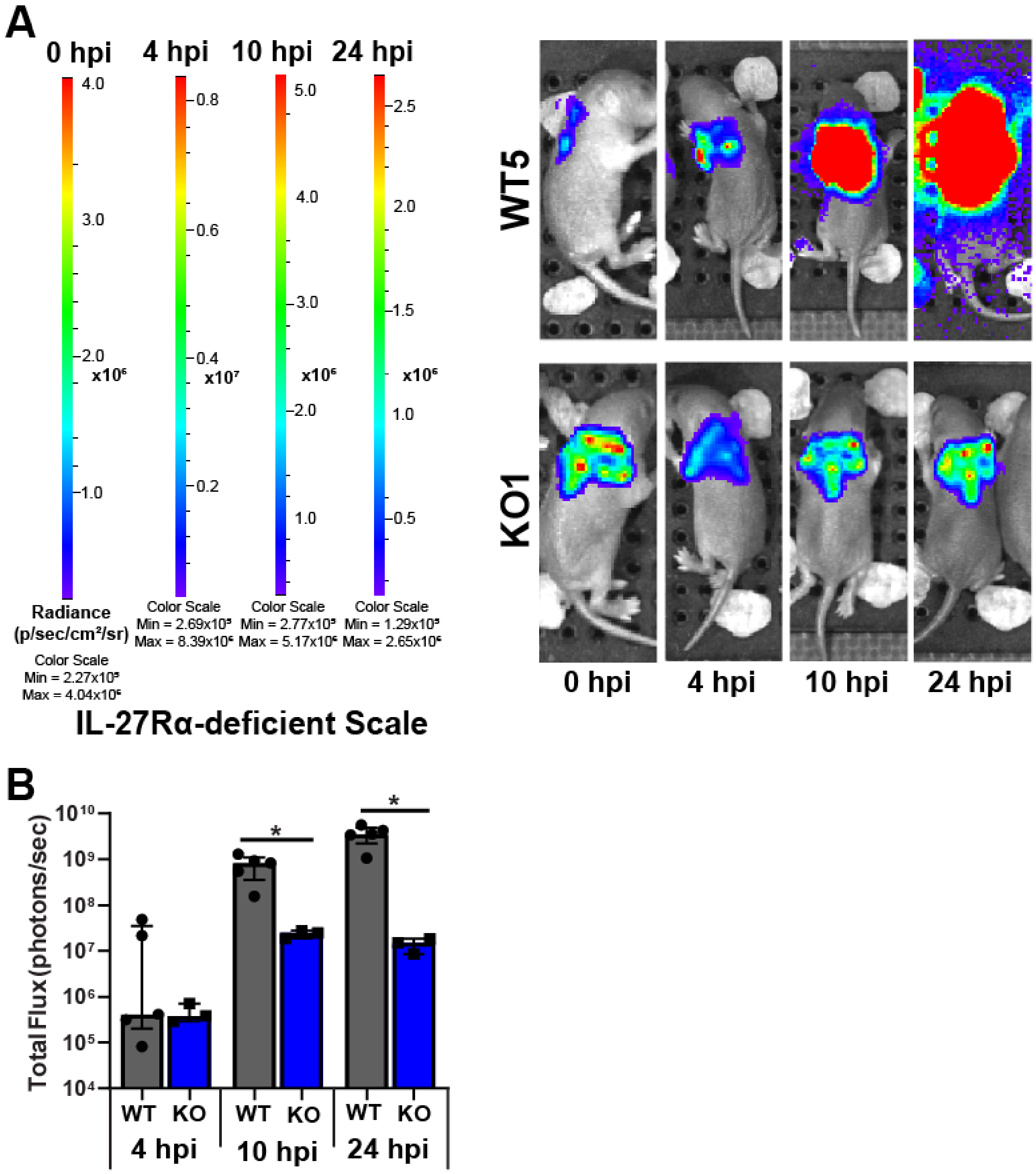

A
0 hpi
4 hpi
10 hpi
24 hpi
Radiance (p/sec/cm^2/sr)
Color Scale
Min = 2.27x10^5
Max = 4.04x10^6
Color Scale
Min = 2.69x10^5
Max = 8.39x10^6
Color Scale
Min = 2.77x10^5
Max = 5.17x10^6
Color Scale
Min = 1.29x10^5
Max = 2.65x10^6
IL-27Rα-deficient Scale
WT5
KO1
B
Total Flux (photons/sec)
WT
KO
4 hpi
10 hpi
24 hpi

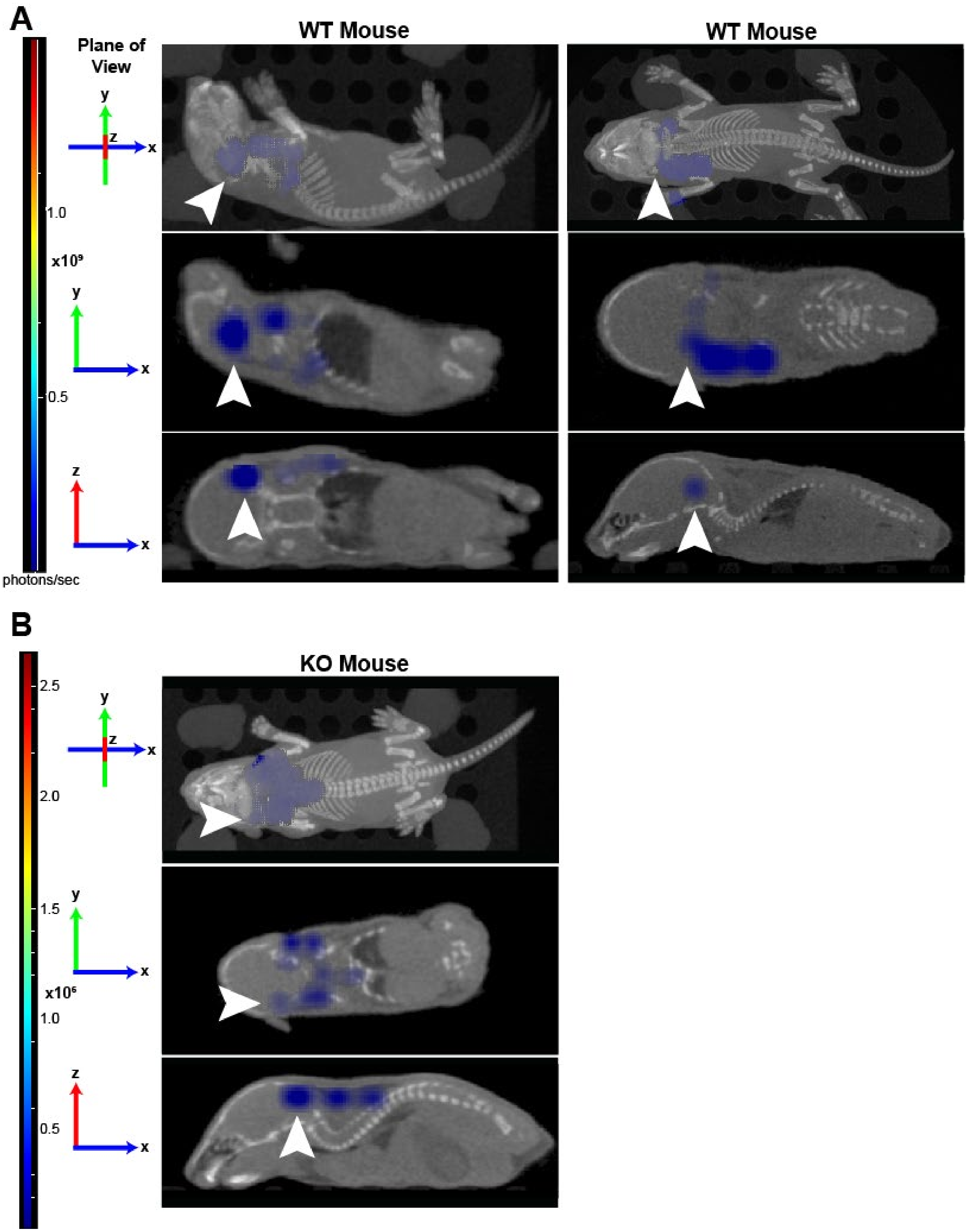
A
Plane of View
WT Mouse
WT Mouse
y
z
x
1.0
x10⁹
0.5
photons/sec
B
KO Mouse
2.5
2.0
1.5
x10⁶
1.0
0.5

Supplemental Figure 1: Cellular profiling of IL-27 producers in the spleen. Neonatal C57BL/6 (WT) mice were subcutaneously inoculated with a target inoculum of ~2x10^6 CFUs/mouse of *E. coli* O1:K1:H7 or PBS as a control on day 3 or 4 of life. At 10 or 24 h post-infection, mice were sacrificed and spleens were harvested. Single cell suspensions of splenocytes were immunolabeled for cell surface markers Gr-1, F4/80, CD11c, or CD115 and intracellular IL-27. Cells were analyzed by flow cytometry. Results from control pups at 10 h (**A**) or 24 h (**B**) are shown. (**C**) Results from infected pups at 24 h; 10 h dot plots were shown in Figure 2.

Supplemental Figure 2: Cellular profiling of IL-27 producers in the blood. Neonatal C57BL/6 (WT) mice were subcutaneously inoculated with a target inoculum of ~2x10^6 CFUs/mouse of *E. coli* O1:K1:H7 or PBS as a control on day 3 or 4 of life. At 10 or 24 h post-infection, mice were sacrificed and blood was collected. Single cell suspensions of PBMCs were immunolabeled for cell surface markers Gr1, F4/80, CD11c, or CD115 and intracellular IL-27. Cells were analyzed by flow cytometry. Results from control pups at 10 h (**A**) or 24 h (**B**) are shown. (**C**) Results from infected pups at 24 h; 10 h dot plots were shown in Figure 3.

Supplemental Figure 3: Live longitudinal imaging of the influence of IL-27 during neonatal sepsis requires separate scales for WT and IL-27Rα$^{-/-}$ mice. Neonatal C57BL/6 (WT) and IL-27Rα$^{-/-}$ (KO) mice were subcutaneously inoculated with a target inoculum of ~2x10^6 CFUs/mouse of luciferase-expressing *E. coli* O1:K1:H7 and imaged longitudinally on an IVIS SpectrumCT at 0, 4, 10, and 24 hours post-infection (hpi). Each mouse was tail-tattooed for individual identification during imaging. Images and data shown are the result of an independent experiment representative of two with similar results (WT, n=5 and KO, n=3). **(A)** Longitudinal

luminescence images of representative WT and IL-27Rα$^{-/-}$ mice at 0, 4, 10, and 24 hpi. Signal is on the KO scale. Colorimetric scale: low (minimum) signal is blue, intermediate signal is green, high (maximum) signal is red. **(B)** Pooled bacterial luminescence signal (total flux) represented as photons/second for all WT or IL-27Rα$^{-/-}$ mice at 4, 10, and 24 hpi. Black circle symbols represent each individual mouse in WT infections, black square symbols represent each individual mouse in KO infections. Statistical analysis of **(B)** was performed using a nonparametric Mann-Whitney U test, median with interquartile range displayed. * = $p \leq 0.05$.

Supplemental Figure 4: Intravital imaging reveals the brain as an organ associated with high bacterial burdens during sepsis. Neonatal C57BL/6 (WT) and IL-27Rα$^{-/-}$ (KO) mice were subcutaneously inoculated with a target inoculum of ~2x10^{6} CFUs/mouse of luciferase-expressing *E. coli* O1:K1:H7 and imaged longitudinally on an IVIS SpectrumCT at 24 hours post-infection (hpi). Images are from an individual experiment representative of two with similar results (WT, n=5 and KO, n=3). **(A)** Representative CT images of two WT mice with bacterial infection in their brains indicated by white arrowheads. Signal is on the WT scale. **(B)** Representative CT images of a KO mouse with bacterial infection in the brain indicated by white arrowheads. Signal is on the KO scale. Perspective (x, y, z), coronal (x, y), and transaxial (x, z) views are shown from 3D CT images for both WT and KO mice. Colorimetric scale: low (minimum) signal is blue, intermediate signal is green, high (maximum) signal is red.
